# Implications of Transcranial Magnetic Stimulation in Geriatric Patients With Pseudodementia

**DOI:** 10.7759/cureus.73981

**Published:** 2024-11-19

**Authors:** Zach Coats, Wayne Coats

**Affiliations:** 1 Osteopathic Medicine, Midwestern University Chicago College of Osteopathic Medicine, Downers Grove, USA; 2 Internal Medicine, Adena Regional Medical Center, Chillicothe, USA

**Keywords:** geriatric psychiatry, major depressive disorder (mdd), pseudo-dementia, transcranial magnetic stimulation (tms), treatment-resistant depression (trd)

## Abstract

This report discusses the case of a female in her 80s with treatment-resistant depression and increasing cognitive decline in recent years. The patient had performed normally on mini-mental status exams (MMSE), had unremarkable CT scans, and been diagnosed with pseudodementia. She had been referred to psychiatry and received multiple courses of different antidepressants and mood-stabilizing medications. However, due to a lack of benefits over time and increasing concern about compliance, she had been referred to a clinic providing transcranial magnetic stimulation (TMS), which had only recently become available in the region. Following a course of 36 treatments over three months, the patient’s cognitive decline resolved, depression symptoms improved, and her family noted that she was back to her normal self.

## Introduction

In recent years, pseudodementia has attracted enormous interest in the medical community due to its complexity and difficulty related to diagnosis. Patients with pseudodementia present with cognitive deficits due to an underlying psychiatric disorder and show symptoms similar to those experiencing dementia [1]. Often, the underlying psychiatric disorder in these patients is major depressive disorder (MDD) [1]. Furthermore, recent studies have shown that the risk of dementia increases by 70% for late-life depressive symptoms [2]. Therefore, when encountering geriatric patients with pseudodementia, the clinical focus should be on effectively treating their depression [1].

Transcranial magnetic stimulation (TMS) is an effective treatment for depression, particularly treatment-resistant depression (TRD). TRD is defined as failure to find benefit despite therapeutic attempts [3]. This classification of depression can be applied to up to 30% of those with MDD and also linked to those with pseudodementia [4]. Depression occurs when the brain produces insufficient neurotransmitters, and TMS uses magnetic pulses to stimulate the structures of the brains of patients with depression [3]. A meta-analysis has reported that patients who receive TMS are more than five times as likely to achieve remission from treatment-resistant depression as patients receiving sham treatment [5].

## Case presentation

The patient was a female in her 80s with a past medical history of Hashimoto’s disease and MDD who had originally presented to her primary care provider (PCP) with worsening symptoms of depression four months after hospitalization following abdominal surgery and isolation during the coronavirus disease 2019 (COVID-19) pandemic. She had subsequently developed a lack of appetite, weight loss (BMI: 19.75 kg/m^2^), reclusiveness, and poor sleep. She had been on citalopram 10 mg on and off for the previous nine years due to compliance challenges.

Upon initial investigation of dementia, the patient had a normal score on the mini-mental status exam (MMSE) (28/30), unremarkable CT, and had only mildly low levels of vitamin B12 of 222 pg/mL (normal range: 250-1,000 pg/mL). She was prescribed supplemental vitamin B12 monthly intramuscular injection of 1000 mcg. At this time, citalopram had been stopped and mirtazapine 15 mg had been initiated with no relief after four months. She had then been referred to psychiatry and prescribed temazepam 15 mg, donepezil 5 mg, and escitalopram 20 mg with some improvement in sleep. At her visit one month after her psychiatry visit, with some improvement in sleep, she appeared even more disinterested, more reserved, and flatter in affect with no attention to self-care or activities of daily living (ADLs). She had returned three months later to assess the effectiveness of the escitalopram, with no relief.

She had been referred for TMS at this time. At her initial consultation for TMS, the patient did not speak or make eye contact, which the providers interpreted as indicative of poor mood and lack of interest, and showed only recent memory recall. At this time, her daughter answered most of the questions. The patient underwent assessment with the Patient Health Questionnaire-9 (PHQ-9) and scored 8/27 (mild depression); however, the providers felt that she was an unreliable historian at this time, and the provider's history showed severe MDD. The patient’s daughter reported that the patient had anhedonia, low mood, isolation, difficulty sleeping, decreased appetite, lack of energy, difficulty focusing, and poor self-esteem coupled with poor self-care. Per history and exam, the patient was diagnosed with pseudodementia and severe MDD. Talk therapy yielded no improvement in symptoms, and hence she was scheduled to undergo treatment with TMS.

Treatment

The patient began a course of 36 TMS treatments over eight weeks all while remaining on the escitalopram. This included five treatments per week for six weeks followed by a taper period of two weeks with three treatments per week. The first treatment included a cranial mapping session to correctly identify the left dorsolateral prefrontal cortex. The patient was seen halfway through treatment (18 sessions) and two months following the end of the 36 treatments. 

Outcome and follow-up

After 18 treatments, the patient still appeared underweight and did not maintain eye contact. However, at this time, the patient’s daughter noted that the patient's depressive symptoms had improved and she was more talkative, showing increased interest in activities, eating better, and playing cards each evening with family. The provider noted a positive change although the patient was still experiencing memory loss, difficulty recalling events, and impaired communication. At this time, her PHQ-9 score was 1/27, indicating no depression, per the patient report with the patient identified as a poor historian. The provider found the patient to have improved symptoms of moderately severe MDD.

After 36 treatments, the patient presented in good condition, and her daughter noted that she seemed to be back to her normal self. She had been more active and was shopping and cooking for herself again; also, she was maintaining interest in social activities, driving, and completing all ADLs; all the while denying symptoms of MDD or dementia. She still appeared underweight; however, she maintained eye contact, had normal speech, was talkative, and had normal memory and attention with a PHQ-9 score of 3/27, indicating no depression. Per history and exam, the patient was euthymic, engaged with family and friends, and performed her ADLs. The patient had also gained weight (BMI: 21 kg/m^2^). The patient attained remission of her MDD and her pseudodementia resolved.

## Discussion

TMS works by stimulating the left dorsolateral prefrontal cortex, which is thought to be an important structure for mood regulation. It has been used to treat depression for over 20 years and was approved by the FDA in 2008. TMS is used primarily in patients who have not received any benefits from first-line antidepressant medications, and it is effective in almost 50% of studied individuals one year following treatment [6]. 

Pseudodementia is also referred to as the dementia syndrome of depression, and this may be a more accurate name for the condition as it often stems from untreated late-life depression with an accompanying decline in cognition. Fortunately, this is treatable and reversible. If this condition is identified and treated effectively, it is possible to avoid neurological damage that could lead to a diagnosis of dementia. However, as there are no diagnostic criteria for pseudodementia, the condition can be challenging to diagnose and treat [7].

There is plentiful literature on the benefits of TMS for depression and the connection between depression and symptoms of dementia (pseudodementia). However, there is scarce data connecting TMS and pseudodementia directly. One systematic review investigated the effect of TMS on mild cognitive impairment (MCI) and found improvements in aphasia and apathy; however, the study had a small sample size, and more evidence through larger-scale trials is required to validate its findings [8]. A literature review focusing on the efficacy of TMS in patients with MCI and those without found it to be similarly effective while reporting an improvement in neurocognitive functioning in both groups (unpublished data). Given this information and the lack of literature connecting TMS to pseudodementia, we can conclude that TMS is an underutilized treatment for pseudodementia.

The limitations of this case report include difficulty in ensuring medication compliance as well as reliance on observations by family members. The patient had difficulties in performing ADLs and providers are unsure of her compliance with previously prescribed antidepressants before her initial decline. The patient remains on escitalopram following her TMS treatment. Furthermore, the reliance on observations by family members may significantly impact the interpretation of the patient’s disease process, thereby impacting the accuracy of the reported symptoms and what the patient was truly thinking and feeling.

## Conclusions

This report highlights the potential benefits of TMS in the treatment of geriatric pseudodementia, particularly in patients with depression. Our patient's improvement in cognition, speech, and ADLs shows that TMS could be potentially life-changing for many patients. Before treatment, our patient's family had considered placing her in long-term care. Today, this patient continues to live independently and free of symptoms of MDD and dementia. While access to TMS clinics continues to improve across the country, many providers are still unaware of the potential benefits of TMS in this population. Further research is needed to establish TMS as a primary treatment method for pseudodementia. If information related to TMS, including mechanisms of action, patient selection criteria, and overall outcomes, can be made widely accessible and integrated into medical education, we believe that many patients can attain benefits similar to the case presented above.
